# A Case of Alpha-1 Antitrypsin Deficiency and Organizing Pneumonia

**DOI:** 10.7759/cureus.12078

**Published:** 2020-12-14

**Authors:** Kavita Renduchintala, Smitha Pabbathi, Sowmya Nanjappa, Asha Ramsakal, John Greene

**Affiliations:** 1 Internal and Hospital Medicine, Moffitt Cancer Center, Tampa, USA; 2 Internal Medicine, Moffitt Cancer Center, Tampa, USA; 3 Infectious Diseases, University of Pittsburgh Medical Center, Pittsburgh, USA

**Keywords:** alpha 1 antitrypsin deficiency, organizing pneumonia

## Abstract

Alpha-1 antitrypsin deficiency (AATD) is an autosomal dominant genetic disorder that presents with pulmonary complications and is most commonly manifested by panacinar emphysema and chronic obstructive pulmonary disease.

A 49-year-old Caucasian female with a history of AATD and chronic tobacco use was referred to both infectious disease and thoracic surgery clinics with worsening cough and chronic intermittent hemoptysis for the evaluation of possible superimposed infection or malignancy. She had previously been treated with multiple antibiotics and Prolastin-CÒ (alpha-1-proteinase inhibitor). Initial CT of the chest showed known chronic bronchiectasis, severe lower lung emphysema, and right-sided lower lobe pulmonary masses. CT-guided biopsy of one mass showed nonspecific inflammation, negative cultures, and negative cytology. Subsequent follow-up with chest CT scans showed a decreasing size of right-sided pulmonary masses and new left-sided nodule formation, which later stabilized in growth. Based on symptoms and radiological and pathological findings, a diagnosis of organizing pneumonia was made.

We present an unusual case of bilateral pulmonary masses mimicking infection and malignancy later found to be most consistent with an organizing pneumonia in a patient with underlying AATD.

## Introduction

Alpha-1 antitrypsin, a 52-kDa plasma protein, is mainly produced in the liver. It is an abundant circulating serine protease inhibitor (serpin). Its main physiological role is to inhibit neutrophil elastase and to contribute as an anti-inflammatory protein [1]. Normally, protease inhibitors counteract the effects of proteases and preserve alveolar structure. This deficiency in protease inhibitor levels gives rise to emphysematous changes. These changes result in a panacinar emphysema often occurring in the lung bases [2]. Lung nodules can also occur in alpha-1 antitrypsin deficiency (AATD). The radiological appearance of such pulmonary nodules can be commonly associated with infections including mycobacterium, such as mycobacterium avium intracellulare, and fungal infections, including aspergillosis, histoplasmosis, and coccidiomycosis. Lung nodules can also occur in both secondary organizing pneumonia and cryptogenic organizing pneumonia (COP), previously known as bronchiolitis obliterans organizing pneumonia [3-5]. Mass-like densities are not typically associated with AATD. When masses are seen on imaging, in addition to the previously discussed causes, malignancy, pulmonary inflammatory diseases with tumor-like lesions, and organizing pneumonia are considered [6].

## Case presentation

A 49-year-old female with a history of chronic bronchitis diagnosed in 1995, AATD diagnosed in 2006, and 25-pack-year prior history of chronic tobacco was referred to infectious disease and thoracic surgery clinics with progressively worsening cough for the evaluation of possible superimposed infection or malignancy. She quit smoking seven years prior to the diagnosis of AATD due to her symptoms of chronic dyspnea and intermittent hemoptysis. She had previously been diagnosed with bronchiectasis and bronchitis for which she had been dependent on oxygen since 1995.

Prior to the clinic visits at our institution, she had been referred to a local pulmonologist and reported taking multiple courses of antibiotics for recurrent pneumonias. These antibiotics included trimethoprim-sulfamethoxazole, azithromycin, doxycycline, levofloxacin, and amoxicillin. She had three prior bronchoscopies, which were negative for bacteria, fungi, and acid-fast bacilli. She had been prescribed a course of prednisone and was already on Prolastin-C® weekly infusions for the diagnosis of AATD. She also underwent pulmonary function tests (PFTs). The FEV1 (forced expiratory volume in one second) after bronchodilator was 1.8 liters, which was 70% of predicted. She had an adjusted DLCO (carbon monoxide diffusing capacity) of 69% of that predicted.

**Figure 1:**
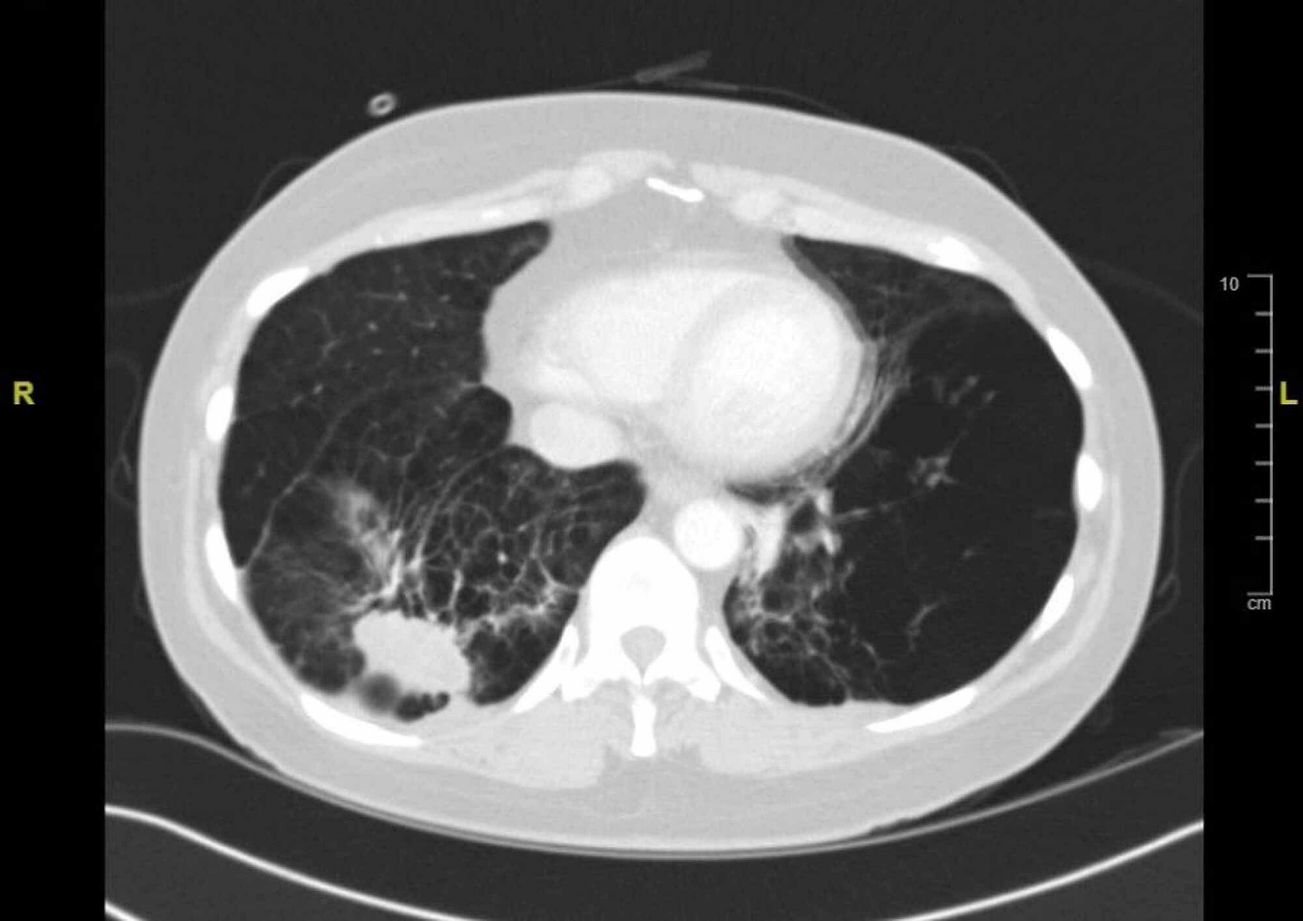
Initial CT of the chest Focal consolidation versus mass in the setting of emphysema is seen in the right lower lung base.

When she presented to the clinic, a high-resolution CT of the chest was ordered for further characterization. The CT (Figure 1) showed development of a mass in the right lower lobe measuring 4.6 cm and another 1.5-cm spiculated nodule slightly more superior, concerning for malignancy. A positron emission tomography (PET) scan was then ordered, which was positive for a 5-cm right lung abnormally hypermetabolic opacity that was concerning for possible infectious process or malignancy.

A CT-guided biopsy was performed on this right lower lobe lung mass for further evaluation. The initial differential diagnosis included infection, malignancy, and noninfectious inflammatory conditions. The cytology was negative for malignancy and showed nonspecific inflammation with histiocytes and also showed necrotic debris. On initial culture results, there was an area of an aggregate of fungal spores. It was unclear at that time if this was a contaminant; therefore, oral voriconazole 200 mg twice daily was started. The gram stain and tissue culture, acid-fast bacteria (AFB) culture, and fungal cultures of the lung biopsy specimen were later determined to be negative, and voriconazole was stopped.

**Figure 2:**
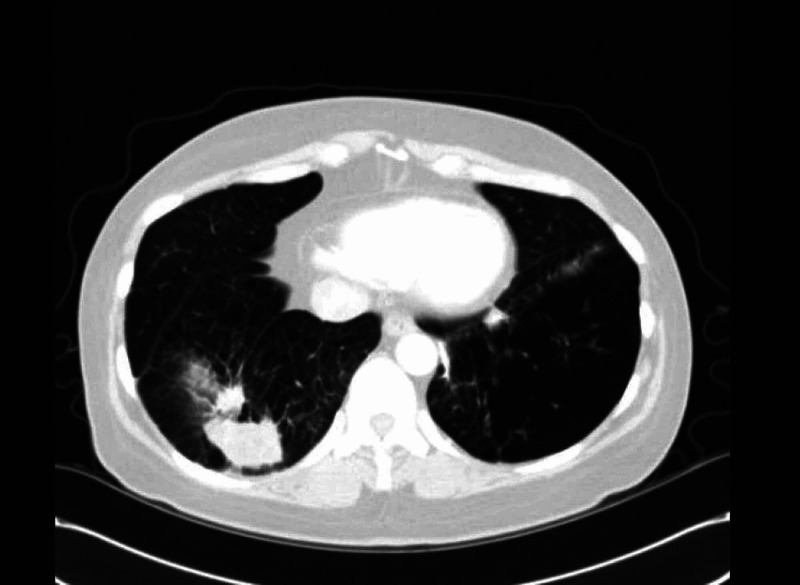
CT of the chest three months later There is persistent right lower lobe lung mass with small mass slightly superior, which has reduced in size compared to prior CT. New left-sided lung nodule was present.

Repeat CT scan (Figure 2) of the chest three months later showed that the 4.6-cm right lower lobe lung mass and the 1.5-cm nodule slightly superior had reduced in size to 4.2 cm and 1.2 cm, respectively. However, there was a new left lower lobe nodule concerning for infection versus inflammation. After reviewing the case with radiology, it was determined that given the development of a new nodule despite prior courses of antibiotics and a recent course of voriconazole, this was unlikely to be an infection but rather represents a diagnosis of an organizing pneumonia. Oral steroids were considered; however, the patient preferred a conservative approach given prior difficulty in tolerating prednisone due to side effects including significant weight gain.

**Figure 3:**
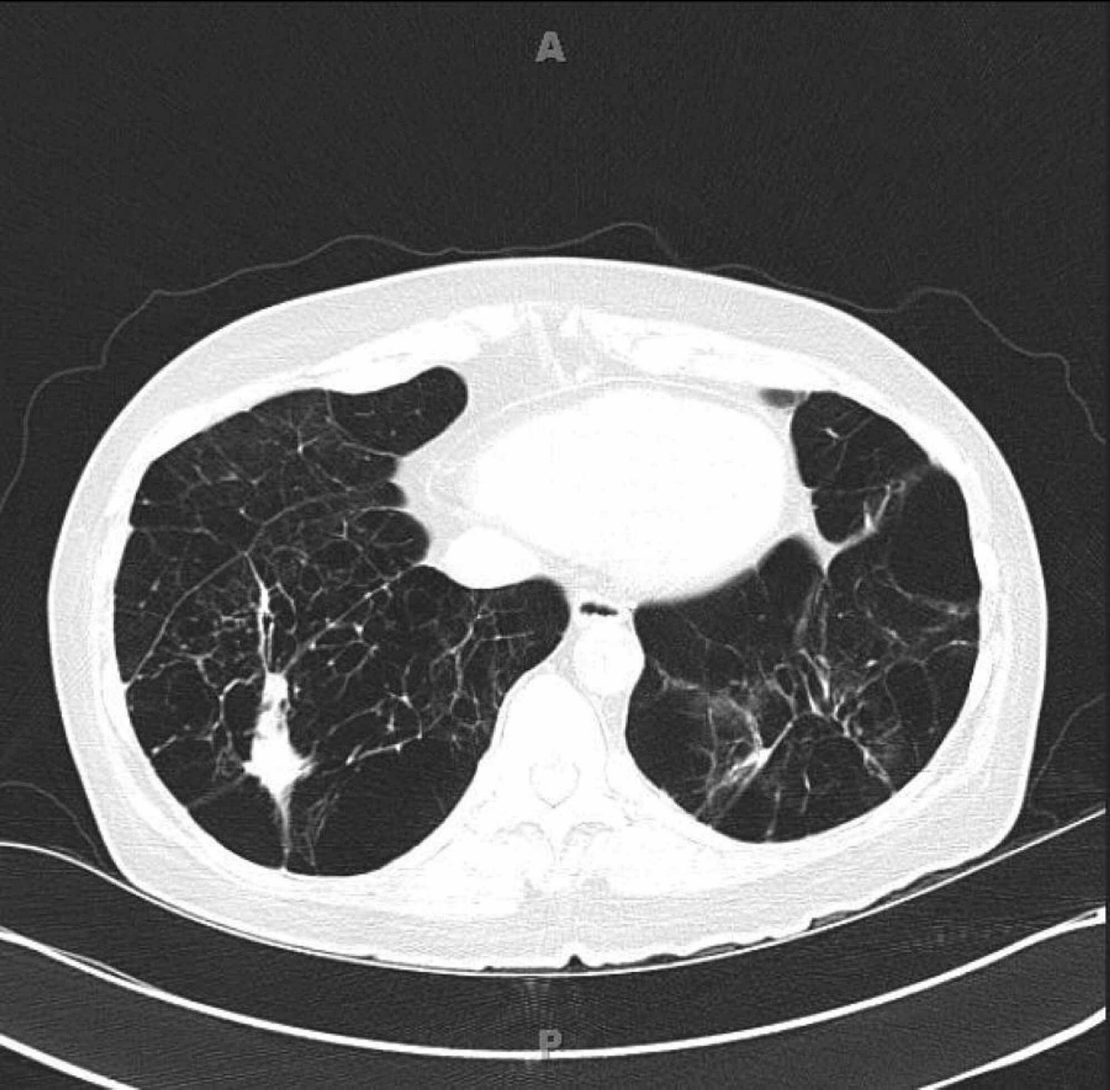
CT of the chest six months later Right lower lung mass is still present but stable in size. Chronic effects from alpha-1 antitrypsin deficiency including known bronchiectatic changes and severe bullous disease of the lower lobes were seen.

Six months later, she reported improvement in symptoms of cough and dyspnea. Repeat CT scan of the chest (Figure 3) showed stable to a slightly larger size of bilateral basilar parenchymal nodules and stable effects from AATD including known bronchiectatic changes and emphysematous disease of the lower lobes. Given that she was doing well off all antibiotics and was no longer having recurrent pneumonia or hemoptysis, it was determined that the bilateral masses were consistent with an organizing pneumonia and should be followed radiologically with no further treatment unless symptoms worsened in the future.

## Discussion

AATD was first described in 1963 by Laurell and Eriksson, who reported an absence of alpha-1 band on protein electrophoresis of serum taken from a patient at a local respiratory hospital [7]. Alpha-1 antitrypsin is produced by hepatocytes. In the lungs, it functions to inhibit neutrophil elastase, a proteolytic enzyme that can destroy alveolar wall connective tissue. The lower the alpha-1 antitrypsin level, the more likely emphysema is to develop. The four main phenotypes are MM (normal), MZ and SZ (heterozygous), and ZZ (homozygous deficiency) [8]. Emphysema is the predominant component of chronic obstructive pulmonary disease (COPD) in AATD. There is also a subgroup of AATD patients who are more likely to develop bronchiectatic changes instead. Treatment of AATD should focus on targeting the enzyme deficiency and the effects of COPD and bronchiectasis [9]. Our patient was getting treated with weekly Prolastin-C infusions.

In regard to obtaining a diagnosis, CT lung densitometry has been demonstrated to be the most sensitive and specific method for the diagnosis of emphysema [10]. PFTs have also been used for many years for monitoring the progression of emphysema [11]. Our patient had been diagnosed with AATD with both CT findings and PFTs prior to her referral to our clinic.

The initial differential for pulmonary nodules or masses in a patient with AATD includes infectious etiologies (bacterial, mycobacterial, fungal), malignancy (primary or metastatic lesions), and other conditions such as sarcoidosis. In our patient, in addition to the infectious workup that was negative, the CT-guided biopsy showed no infectious or malignant etiology for these masses. The patient’s symptoms of cough, dyspnea, and hemoptysis, along with the pulmonary masses on imaging and the lung biopsy findings of nonspecific inflammation with histiocytes and necrotic debris, were consistent with a diagnosis of organizing pneumonia, which is not typically associated with AATD. Organizing pneumonia is divided into two categories. The first category is a COP and the second category is a secondary organizing pneumonia.

COP, previously known as bronchiolitis obliterans organizing pneumonia, is a rare but well-defined disorder that is characterized by the patchy involvement of air spaces by small polypoid tufts of organizing connective tissue distributed within terminal bronchioles, alveolar ducts, and alveoli [12]. The main radiological finding of COP as seen on chest CT scan is bilateral multifocal consolidation that can be migratory. Another manifestation of COP is that of solitary pulmonary nodule or mass that can simulate carcinoma, leading to biopsy or resection [13]. This manifestation was what was seen in our patient. Pulmonary ground-glass opacity nodules on CT scan can represent as organizing pneumonia; however, biopsy should be considered as this can also represent malignancy [14]. Our patient’s biopsy showed nonspecific inflammation with histiocytes and necrotic debris, which is consistent with an organizing pneumonia.

There are various types of COP including typical COP, acute fulminant COP, fibrosing COP, and unifocal COP. In typical COP, spontaneous remission can occur in 50% of the cases. The other 50% of the cases are treated with low-dose corticosteroid treatment. In acute fulminant COP, patients present with rapid onset acute respiratory distress syndrome, and oral or intravenous corticosteroid therapy is needed. Fibrosing COP presents with fibrosis on biopsy. Unifocal COP presents with a solitary pulmonary nodule that usually does not recur if removed. Treatment for all types of COP is based on clinical presentation. If a patient has mild disease and typical COP, spontaneous remission can occur. However, corticosteroids are often used. In severe cases, medications such as azathioprine, cyclophosphamide, or cyclosporine are needed [15].

Secondary organizing pneumonia can occur due to lung injury from causes such as infection, drug toxicity, autoimmune disorders, and radiotherapy, none of which our patient had. It can also be associated with certain lung pathologies including vasculitis, malignancy, eosinophilic pneumonia, pulmonary infarct, and interstitial lung diseases. Our patient did not have any of these diagnoses. Secondary organizing pneumonia can present in a nodular form, which includes solitary or multiple nodules or masses. It can also present as a fluctuating and migrating multifocal parenchymal consolidation. The treatment of any secondary organizing pneumonia is to first treat the underlying cause and then add steroids if needed. Interestingly, even though AATD is known to affect the lungs, it is not included in reviews and tables discussing causes of secondary organizing pneumonia [15-17].

Pulmonary involvement of AATD generally involves panacinar emphysema, bronchiectasis, COPD, and asthma. Notably, organizing pneumonia is not typically associated with AATD. However, the presentation of our patient suggests the coexistence of both AATD and an organizing pneumonia.

Classifying any organizing pneumonia as secondary or cryptogenic requires thorough review of the patient’s clinical history including medication exposure and past medical history. Our patient did not have any causes typically associated with secondary organizing pneumonia and had spontaneous remission of her pulmonary masses, which is consistent with a diagnosis of typical COP. In AATD, the deficiency of alpha-1 antitrypsin leads to alveolar damage due to the lack of protease inhibition. In organizing pneumonia, the distal bronchioles, alveolar ducts, and alveoli are affected. It is possible that the deficiency of alpha-1 antitrypsin can make patients more susceptible to an organizing pneumonia due to underlying alveolar damage. Further studies are needed to determine if AATD can be considered as an underlying cause for a secondary organizing pneumonia.

## Conclusions

In conclusion, the patient in our report had an unusual radiological pattern of pulmonary masses that mimicked infection or malignancy, which would have been the most likely diagnoses given her history of AATD. However, she was later found to have clinical course and imaging consistent with an organizing pneumonia. Organizing pneumonia should be considered in the differential diagnosis of patients with AATD and pulmonary masses.
